# PSMA expression by microvasculature of thyroid tumors – Potential implications for PSMA theranostics

**DOI:** 10.1038/s41598-017-05481-z

**Published:** 2017-07-12

**Authors:** Andrey Bychkov, Usanee Vutrapongwatana, Supatporn Tepmongkol, Somboon Keelawat

**Affiliations:** 1 Department of Pathology, Faculty of Medicine, Chulalongkorn University, Bangkok, 10330 Thailand; 2 Division of Nuclear Medicine, Department of Radiology, Faculty of Medicine, Chulalongkorn University, Bangkok, 10330 Thailand

## Abstract

Prostate-specific membrane antigen (PSMA) is overexpressed in prostate cancer epithelium, making it a promising target for molecular imaging and therapy. Recently, several studies found unexpected PSMA radiotracer uptake by thyroid tumors, including radioiodine-refractory (RAIR) cancers. PSMA expression was reported in tumor-associated endothelium of various malignancies, however it has not been systematically addressed in thyroid tumors. We found that PSMA was frequently expressed in microvessels of thyroid tumors (120/267), but not in benign thyroid tissue. PSMA expression in neovasculature was highly irregular ranging from 19% in benign tumors to over 50% in thyroid cancer. Such heterogeneity was not directly attributed to endothelial cell proliferation as confirmed by immunostaining with proliferation-associated endothelial marker CD105. PSMA expression was associated with tumor size (p = 0.02) and vascular invasion in follicular carcinoma (p = 0.03), but not with other baseline histological, and clinical parameters. Significant translational implication is that RAIR tumors and high-grade cancers maintain high level of PSMA expression, and can be targeted by PSMA ligand radiopharmaceuticals. Our study predicts several pitfalls potentially associated with PSMA imaging of the thyroid, such as low expression in oncocytic tumors, absence of organ specificity, and PSMA-positivity in dendritic cells of chronic thyroiditis, which is described for the first time.

## Introduction

Prostate specific membrane antigen (PSMA) is a type II transmembrane glycoprotein highly restricted to prostate epithelium^1, 2^. It is also known as FOLH1 (folate hydrolase 1) or glutamate carboxypeptidase II. Immunohistochemical studies reported that PSMA is strongly expressed by normal and neoplastic prostatic epithelium, along with the epithelium of other genitourinary organs (bladder, kidney, fallopian tubes) and intestine^3–5^. Several recent studies found that PSMA could be expressed not only by epithelial cells, but also by vascular endothelium of various malignancies including oral^6^, gastric and colorectal^7^, lung^8^, breast^9^, endometrial and ovarian^10^, renal^11^, urothelial^12^, and glial tumors^13, 14^.

PSMA is an integral membrane protein, anchored to the epithelial cells. This makes an important advantage of being a targeting marker over prostate-related secretory antigens released into bloodstream, such as prostate specific antigen, prostatic acid phosphatase and prostate secretory protein^15^. Molecular imaging of PSMA is now being widely adopted in prostate cancer diagnostics^16–18^. ^68^Ga-PSMA PET/CT is a novel imaging modality based on ^68^Ga conjugated with anti-PSMA monoclonal antibody, which is highly accurate in detecting prostate cancer^19^. It also has promising therapeutic potential, being a carrier for radionuclides directed against cancer cells. One of such theranostic example is ^177^Lu-labelled tracer PSMA-DKFZ-617, which demonstrated radiological response in preclinical model and clinical studies of prostate cancer^18, 20^. Targeted α-therapy with ^225^Ac-PSMA was able to shift patients with advanced metastatic prostate cancer into complete remission^21^.

Wide use of ^68^Ga-PSMA PET/CT for prostate imaging yielded a plethora of reports with unexpected detection of non-prostate tumors, including primary and metastatic breast, renal, neuroendocrine and other malignancies^17, 22^. Several consecutive case reports described ^68^Ga-PSMA-positive thyroid tumors, including adenoma^23–25^ and carcinoma^26–28^. Many of these tumors were detected incidentally in patients screened for prostate cancer. A growing number of such reports reflects increased use of ^68^Ga-PSMA PET/CT and also high incidence of thyroid nodules in the population. It is widely acclaimed that thyroid cancer today is the fastest growing malignancy in the developed world^29^. Prognosis in a majority of patients is excellent due to high efficacy of treatment based on thyroidectomy followed by radioiodine (RAI) ablation of thyroid remnants. However up to 15–20% of all thyroid cancers may lose their ability to trap RAI, hence they are being hidden for RAI imaging and therapy^30^. RAI-refractory thyroid cancer, whether local or disseminated, requires alternative imaging strategies, usually with ^18^FDG PET/CT, which is still unable to detect all the foci. It is compelling that ^68^Ga-PSMA PET/CT imaging can potentially identify RAI-refractory metastases in patients with negative RAI scan, and serve as a potential therapeutic opening.

PSMA expression has not been systematically studied in thyroid tumors, and only limited evidence is available about absence of PSMA expression in benign thyroid^4, 31^. Tissue microarray study found occasional and weak PSMA expression in less than 5% of follicular and papillary thyroid cancers^4^. One recent report described ^68^Ga-PSMA PET/CT-positive follicular thyroid adenoma with PSMA expression in tumor neovasculature endothelial cells, but not in thyroid epithelial cells^24^. Aberrant expression of PSMA in thyroid carcinoma may have a potential to widen therapeutic options in the management of RAI-refractory thyroid cancer. A study of a large cohort of thyroid tumors would be desirable to further elucidate these issues and potentially advocate the use of PSMA-based imaging in thyroid patients^32^.

We aimed to evaluate PSMA expression by immunohistochemistry and to perform clinicopathological correlation in a wide range of thyroid tumors, including a cohort of RAI-refractory thyroid cancer. Our study outlines possible implications and also pitfalls that can be encountered during evaluation of PSMA-based imaging in both thyroid and non-thyroid patients.

## Results

### PSMA expression in thyroid

**Figure 1:**
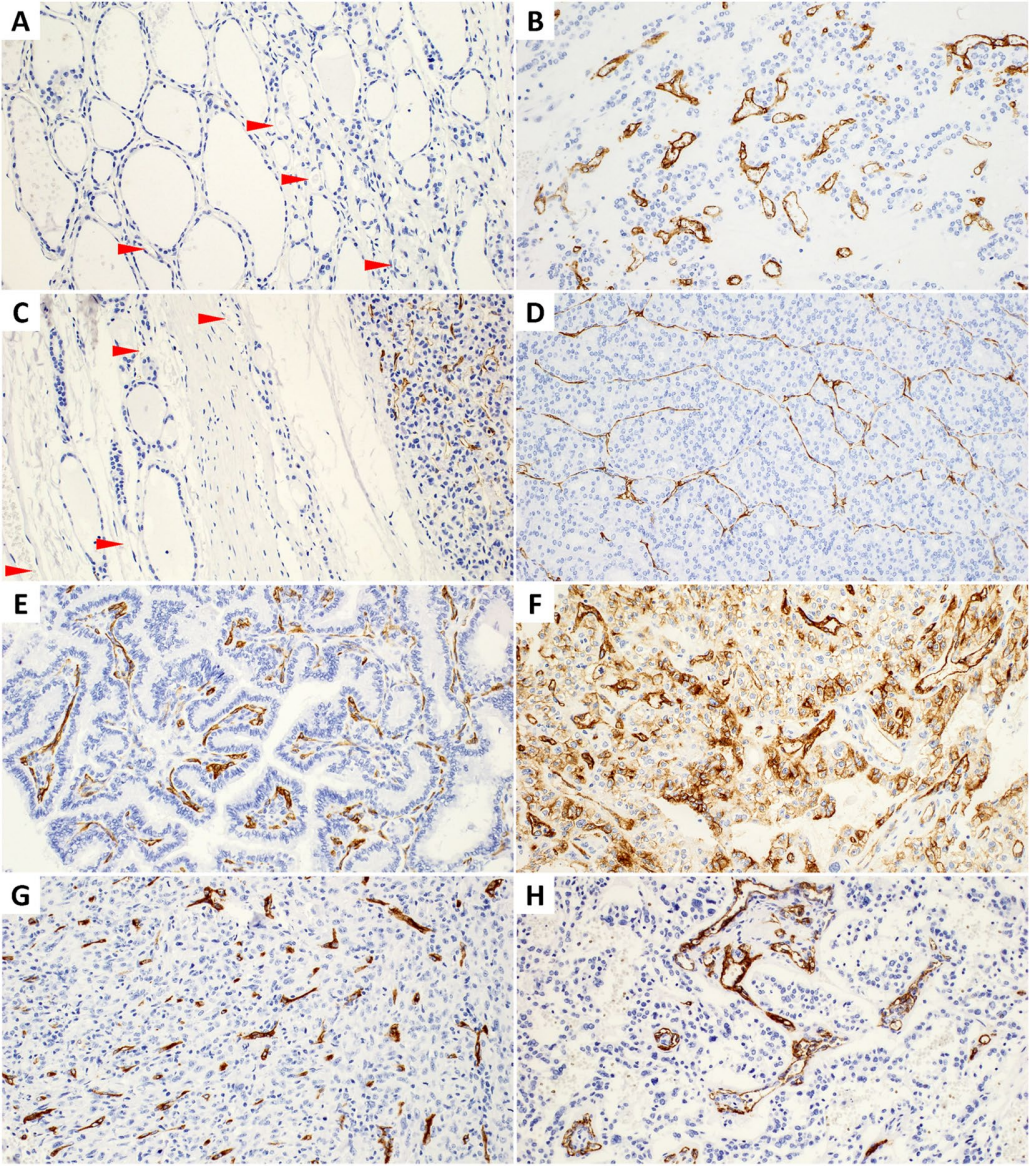
PSMA immunoreactivity in representative thyroid samples. PSMA is expressed by endothelium of thyroid tumors (**B**–**H**), but not of benign thyroid (**A**,**C**). Arrowheads indicate microvessels with red blood cells in non-neoplastic thyroid tissue. Note absence of PSMA expression by benign or neoplastic epithelium, except one case of PDTC (**F**). (**A**) Benign thyroid. (**B**) Follicular adenoma. (**C**) Interface between follicular cancer and adjacent thyroid tissue. PSMA expression is restricted to the tumor microvasculature. (**D**) Follicular thyroid carcinoma. (**E**) Papillary thyroid carcinoma, classic variant. PSMA-positive capillaries decorate papillary cores. (**F**) Poorly differentiated thyroid carcinoma with ectopic PSMA expression by cancer cells added by predictable immunoreactivity in tumor-associated endothelium. (**G**) Anaplastic thyroid carcinoma. (**H**) Medullary thyroid carcinoma. Immunohistochemistry, 200× (**A**–**H**).

Only one case out of 267 neoplastic and 191 non-neoplastic thyroid samples showed aberrant epithelial expression of PSMA. It was a poorly differentiated thyroid carcinoma (PDTC) displaying cytoplasmic immunoreactivity with membranous accentuation in approximately 20% of cancer cells (Fig. 1F). In all the remaining cases, normal and neoplastic (whether benign or well-differentiated malignant) follicular and parafollicular thyroid cells did not express PSMA (Fig. 1A–E,G,H). At the same time we observed frequent PSMA immunoreactivity of endothelium of tumor microvasculature (Fig. 1B–H), which is the most likely explanation of positive PSMA imaging of various thyroid tumors reported in the literature.

Endothelial PSMA staining was heterogeneous among different thyroid tumors (Table 1). Briefly, PSMA in tumor capillaries was expressed in 19% follicular adenomas (FA), 46% follicular carcinomas (FTC), 51% papillary carcinomas (PTC), and 40–50% high-grade thyroid cancers. Most often, PSMA immunoreactivity was noted in RAI-resistant thyroid cancer (63% cases). These findings suggest that endothelial PSMA expression increases in parallel with tumor progression. Indeed, there was a statistically significant difference in PSMA score in pairs of FA-FTC (p = 0.01, U test) and FA-PTC (p < 0.01, U test). Moreover, PTC follicular variant demonstrated lower PSMA score compared to PTC classic variant (p = 0.03, U test), but not different from FTC (p = 0.67, U test). Oncocytic tumors (FA and FTC) had lower endothelial expression of PSMA than their non-oncocytic counterparts (p = 0.04, U test). There was no statistically significant difference of PSMA expression among FTC, PTC, RAI-refractive cancer and high-grade tumors (p = 0.58, Kruskal-Wallis test).

**Table 1:** PSMA expression in the microvessels of thyroid tumors.

	Total	PSMA +ve		mean PSMA	Low PSMA (5–50%)		High PSMA (>50%)	
	*n*	*n*	%	proportion score	*n*	%*	*n*	%*
Follicular adenoma	43	8	19%	3.7	8	100%	0	0%
Non-oncocytic	32	7	22%	5.3	7	100%	0	0%
Oncocytic	11	1	9%	1.3	1	100%	0	0%
Follicular thyroid carcinoma	52	24	46%	10.6	19	79%	5	21%
Non-oncocytic	35	20	57%	11.6	16	80%	4	20%
Oncocytic	17	4	24%	7.4	3	75%	1	25%
Papillary thyroid carcinoma	120	61	51%	24.7	33	54%	28	46%
Classic variant	49	29	59%	30.0	12	41%	17	59%
Follicular variant	33	12	36%	13.7	6	50%	6	50%
Tall cell variant	5	4	80%	44.4	2	50%	2	50%
Microcarcinoma	9	3	33%	16.0	2	67%	1	33%
Lymph node metastasis	24	13	54%	11.6	11	85%	2	15%
RAI-refractory carcinoma	24	15	63%	32.5	8	53%	7	47%
Poorly differentiated carcinoma	8	4	50%	37.0	2	50%	2	50%
Anaplastic thyroid carcinoma	10	4	40%	21.0	1	25%	3	75%
Medullary thyroid carcinoma	10	4	40%	13.7	3	75%	1	25%
Total	267	120	45%	17.9	74	62%	46	38%

*Per cent out of all PSMA-positive cases within particular tumor type.

**Figure 2:**
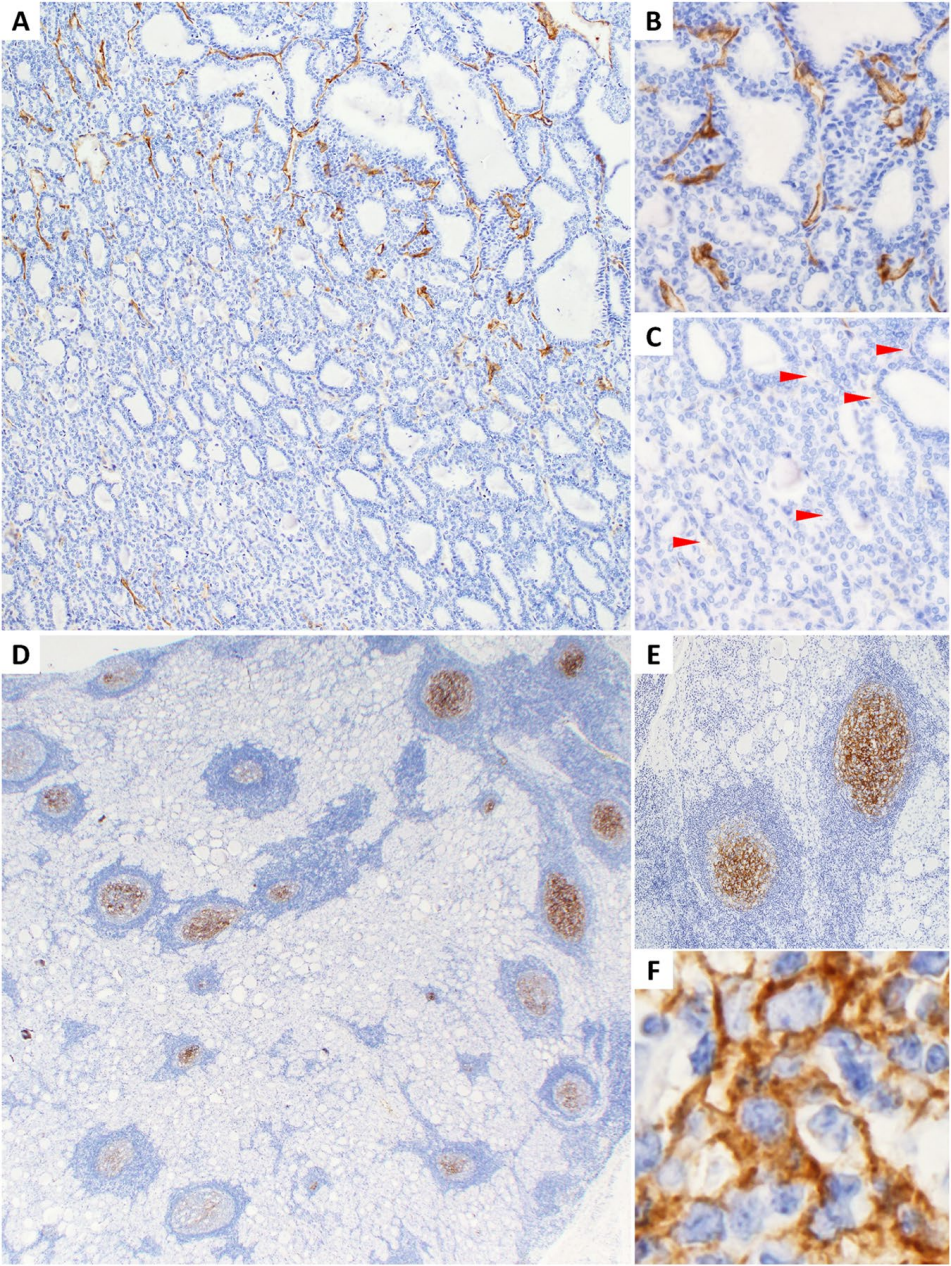
Potential imaging pitfalls associated with PSMA expression in thyroid lesions. (**A**) Significant heterogeneity within follicular carcinoma. Two neighboring areas with high (**B**) and negative (**C**) PSMA expression in tumor capillaries. Arrowheads indicate PSMA-negative microvessels. (**D**) Hashimoto’s thyroiditis with prominent PSMA immunoreactivity in lymphoid follicles (**E**) due to cytoplasmic staining of dendritic cells (**F**). Note absence of PSMA-positive endothelium. Immunohistochemistry, 100× (**A**,**E**), 400× (**B**,**C**), 20× (**D**), 800× (**F**).

Endothelial expression of PSMA was exclusively localized within tumors, but not in the normal or non-neoplastic (e.g., multinodular goiter) thyroid. Prominent capillary network of benign thyroid disclosed by CD31/CD34 immunostaining or intravascular red blood cells was consistently negative for PSMA (Fig. 1A,C). Intratumoral distribution of PSMA-positive microvessels was variable, being mainly associated with capillaries, and rarely with larger vessels. Heterogeneity of PSMA expression could be easily appreciated within one section (Fig. 2A–C). Spatial distribution was often restricted to certain tumor areas, however no correlation with tumor pattern was noted. Encapsulated tumors (FA, FTC, and PTC) tended to express PSMA in central zone. Furthermore, no association was observed between PSMA expression and invasive front of infiltrative cancers. Among all endothelial PSMA-positive thyroid tumors, the highest expression according to proportion score was in anaplastic cancer (ATC) and PDTC. There were five cases of PTC with matched metastases, and only three of them (all PSMA-negative) showed concordance between primary and secondary tumors. The remaining two PSMA-positive primary cancers developed PSMA-negative metastases.

Surprisingly, lymphoid follicles of Hashimoto’s thyroiditis displayed PSMA expression (Fig. 2D–F). This aberrant positivity was attributed to dendritic cells (confirmed with specific markers of dendritic cells CD21 and CD23), but not microvasculature (ruled out by CD31 and CD34 staining). Cytoplasm and processes of dendritic cells showed PSMA immunoreactivity (Fig. 2F). All 30 cases of Hashimoto’s thyroiditis had at least one PSMA-positive lymphoid follicle. After counting all lymphoid follicles in Hashimoto’s samples we found that 48% (293/610) of the follicles were PSMA-positive. Dendritic cells also maintained PSMA expression in intratumoral lymphoid follicles of Warthin-like PTC (2 cases).

### Correlation with endothelial markers

Pilot staining of 50 samples with variable PSMA expression including FA (n = 5), FTC (n = 10), PTC (n = 25), PDTC (n = 4), ATC (n = 3), and medullary thyroid carcinoma (MTC; n = 3) with CD31/CD34 and further matching, demonstrated that PSMA was not ubiquitously expressed in tumor microvasculature. Microvessel density, scored with automatically Aperio Imagescope Microvessel Analysis algorithm, found no correlation between CD31/CD34 and PSMA vascular network (p > 0.5, Spearman’s correlation). As it has been shown above, only 45% of thyroid tumors from our large set were PSMA-positive (Table 1). Furthermore, PSMA expression in 90–100% of tumor microvessels was observed only in 9 out of 267 tumors (3.4%).

**Figure 3:**
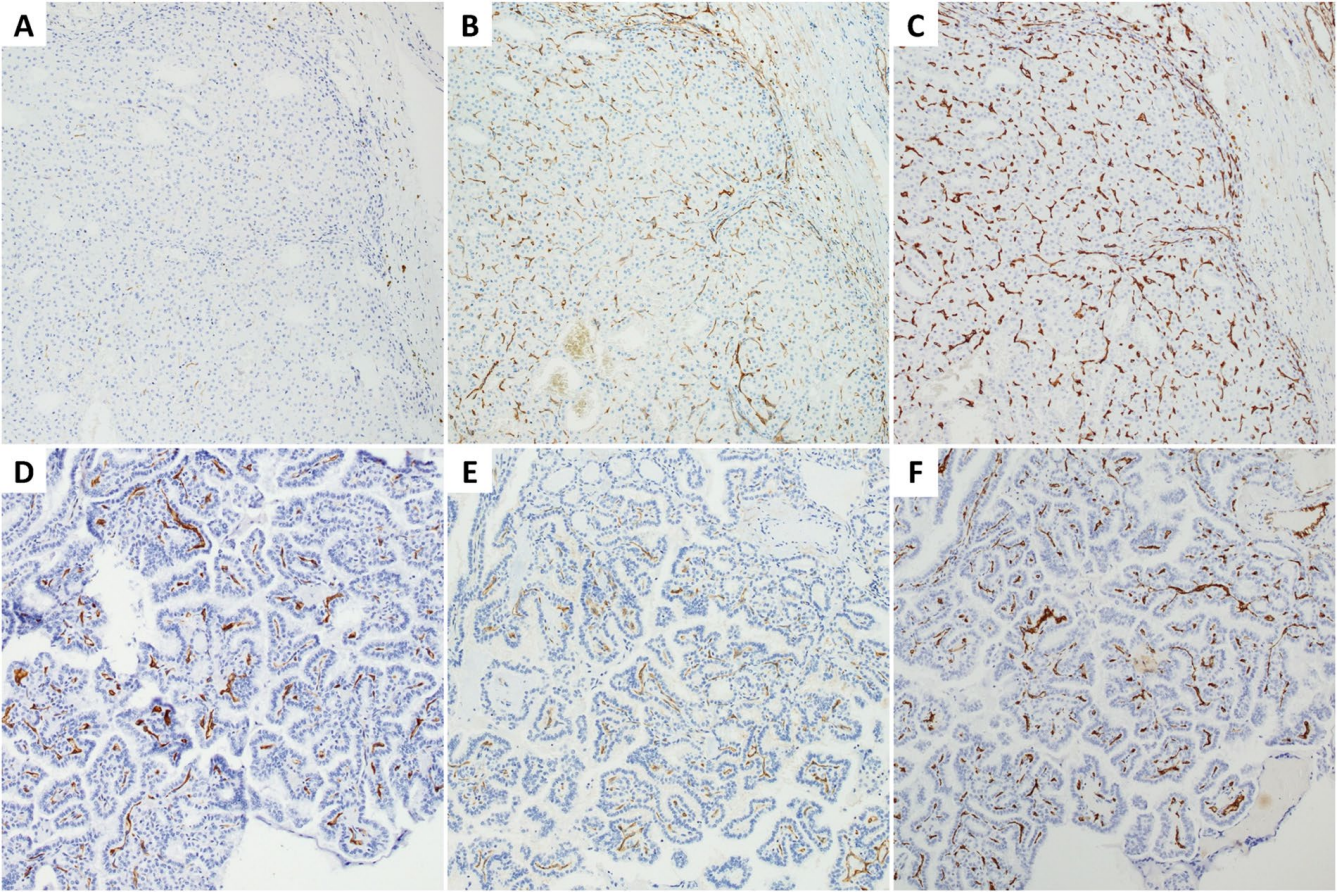
Coexpression of PSMA and endothelial markers. (**A**–**C**) Follicular thyroid carcinoma with negative expression of PSMA (proportion score <5%) versus strongly positive CD105 and CD34. (**D**–**F**) Papillary thyroid carcinoma with concordant expression of PSMA, CD105, and CD34. Immunohistochemistry with anti-PSMA (**A**,**D**), anti-CD105 (**B**,**E**), and anti-CD34 (**C**,**F**). Serial sections, 100×.

To address this issue we hypothesized that PSMA expression might be associated with proliferating endothelium. The same set of 50 samples was stained with a marker of vascular endothelial cell proliferation CD105. CD34 was used as a reference marker highlighting 100% of microvasculature. CD105 was rarely expressed in benign thyroid, getting more prominent in hyperplastic nodules. At variance, all thyroid tumors displayed endothelial expression of CD105, however no complete concordance with CD34 was noted. Mean CD105 score for studied samples was 73.4 (range 5–100), whereas mean PSMA score – 28.9 (range 0–100). Manual and automated scoring found no significant correlation between PSMA and CD105 expression (R^2^ = 0.08, p = 0.14, Pearson correlation). Indeed, only half out of 50 specimens showed comparable scores of CD105 and PSMA (Fig. 3A–C). The rest demonstrated higher (23/50) or lower (2/50) expression of CD105 (Fig. 3D–F). We concluded that intratumoral heterogeneity of PSMA immunostaining is not directly related to endothelial cell proliferation.

### Clinical significance of endothelial PSMA expression in thyroid tumors

Correlation analysis revealed that PSMA expression was significantly associated with size of thyroid carcinoma (R^2^ = 0.3, p = 0.02, Spearman’s correlation). This is an important prognostic indicator of thyroid cancer, which also hold true for FTC and PTC, separately. Among histopathological characteristics, oncocytic phenotype of FA had association with lower PSMA score compared to non-oncocytic variant (p = 0.03, chi square test). Higher score in FTC was associated with vascular invasion (p = 0.03, chi square test). Other baseline (age, gender), histopathological (extrathyroidal extension, positive margin), and clinical (nodal and distant metastasis, AJCC/UICC stage, MACIS score) parameters, either in FTC, PTC, or in different PTC variants, were not associated with PSMA score (Table 2).

**Table 2:** Relationship of PSMA expression to clinicopathological parameters in well differentiated (primary papillary and follicular) thyroid carcinomas.

		PSMA-positive	PSMA-negative	p-value^a^
		*n* = 72	*n* = 76	
Age, mean ± SD (range), years		44.8 ± 18.1	47.6 ± 14.6	0.31
Sex	Male	17	15	0.55
	Female	54	62	
Tumor size, mean ± SD (range), mm		40.7 ± 24.7	31.3 ± 16.1	0.01
Positive surgical margin		14	8	0.16
Extrathyroidal extension		22	17	0.27
Capsular/vascular invasion*		24/32	30/42	0.79
PTC variant	Classic	29	20	0.07^b^
	Follicular	12	21	
	Tall cell	4	1	
	Microcarcinoma	3	6	
T category (1 + 2 vs. 3 + 4)	1	10	12	0.14
	2	21	30	
	3	38	34	
	4	3	0	
N category**	0	39	38	
	1	9	10	1.00
M category	0	69	74	
	1	3	2	0.67
Clinical stage (I + II vs. III + IV)	I	35	41	0.15
	II	11	16	
	III	21	16	
	IV	5	3	
MACIS score, mean ± SD (range)		5.4 ± 1.9	5.1 ± 1.4	0.23

^a^Two-tailed values based on t-test for continuous variables and Fisher’s exact test for categorical variables. ^b^Chi square test. *Only encapsulated tumors. **Only papillary cancers.

### PSMA expression in non-thyroid tissues

PSMA was consistently expressed by prostate cancer epithelium, and also by endometrial adenocarcinoma (Table 3). Among all the non-neoplastic samples studied, epithelial expression of PSMA was detected only in the renal tubules. Similar to the cases of Hashimoto’s thyroiditis, we could observe frequent and strong expression of PSMA in dendritic cells of benign cervical lymph nodes (7 out of 12 nodes).

**Table 3:** PSMA immunoreactivity in non-thyroid tissues.

	Localization	
	Endothelial	Other
Normal		
Lymph node	0/12	7/12 (dendritic cells)
Parathyroid	0/3	0/3
Thymus	0/2	0/2
Lung	0/1	0/1
Kidney	0/2	2/2 (epithelium)
Urether	0/1	0/1
Prostate	0/2	0/2
Malignant		
Prostate	2/4	4/4 (epithelium)
Kidney	1/3	0/3
Uterus	1/1	1/1 (epithelium)
Ovary	1/1	0/1
Breast	3/4	0/4
Lung	3/3	0/3
Breast	3/4	0/4
Stomach	1/2	0/2
Colon	2/4	0/4
Pancreas	1/2	0/2
Liver	1/1	0/1

Regarding endothelial expression, there was no PSMA immunoreactivity in microvasculature of various normal organs (Table 3). However, wide range of malignant tumors showed heterogeneous expression of PSMA in cancer microvessels, comparable in scores with thyroid carcinoma. This finding points out that PSMA expression in cancer-associated microvasculature has universal rather than organ-specific pattern.

## Discussion

This is the first comprehensive study of PSMA expression in a wide range of thyroid specimens. Our results indicate that PSMA immunoreactivity is frequent in thyroid tumors, being attributed to endothelial, but not epithelial expression. Important translational implication of the findings is that thyroid cancer microvasculature may be a promising target for PSMA-directed treatment, especially in the subsets of RAI-refractory and aggressive high-grade thyroid carcinomas. However, PSMA-reactivity is not specific to thyroid cancer, which can be a pitfall for PSMA-targeted imaging and therapy in thyroid practice.

Our study found that PSMA in thyroid is expressed by endothelium of tumor-associated microvessels. No cancer cells except one case of PDTC were immunoreactive for PSMA. No normal thyroid epithelium or non-neoplastic microvasculature were PSMA-positive, which is in the line with the recent biodistribution study that found minimal uptake of ^68^Ga-PSMA-11 PET/CT radiotracer in thyroid in 56 patients compared to significantly higher uptake in thyroid nodules^32^. PSMA expression in neovasculature was observed in a wide spectrum of thyroid tumors from benign adenomas to highly-aggressive undifferentiated carcinomas. Cancers had significantly higher PSMA expression than benign tumors (p < 0.01, Fisher’s exact test).

An important finding, which may have significant translational implication, is that high-grade RAI-negative cancers, such as PDTC and ATC, and also MTC demonstrated striking endothelial expression of PSMA. PSMA-based theranostic applications can be a promising modality in these aggressive cancers. In fact, Verburg *et al*. were able to visualize metastatic RAI-negative PDTC by PSMA PET/CT^28^. On the other hand, a fair amount of benign FA (19%) and indolent subcentimeter cancers, i.e. microcarcinomas (33%), were PSMA-positive. The recent case report described 4 mm FTC incidentally detected by PSMA imaging^26^. A resolution of PSMA PET/CT is high enough to recognize 2.4 mm sized metastatic prostate cancer^17^. It is important to be aware that PSMA expression is not restricted only to true thyroid malignancies to avoid misinterpretation while evaluating thyroid lesions on ^68^Ga-PSMA ligand PET/CT^24^.

Ability to recognize non-RAI-avid thyroid cancer is a major advantage of PSMA-based imaging. We found that RAI-refractory recurrent and metastatic PTC expressed PSMA even more often than unrelated primary PTC (63% *vs*. 51%). Several publications reported about efficiency of ^68^Ga-PSMA PET/CT in recognition of various RAI-resistant thyroid cancers, including PTC, FTC, and PDTC^27, 28, 33^. ^18^F-FDG PET/CT is one of the most sensitive imaging modalities currently used in patients with RAI-refractory thyroid cancer^30^. Preliminary clinical reports found that ^68^Ga-PSMA imaging might have benefit over ^18^F-FDG PET/CT, because it tends to detect more metastatic RAI-resistant foci, and was more effective in recognition of brain lesions^27, 33^. Similar results were also observed in prostate cancer, where PSMA PET/CT appeared more sensitive than conventional ^18^F-FCH PET/CT^20^. PSMA targeting in thyroid cancer holds great promise not only as alternative imaging modality for detection and staging of RAI-refractory carcinoma, but also as a potential theranostic application. ^177^Lu-PSMA-based radionuclide therapy has been tested in prostate cancer, and is currently considered for trials in RAI-resistant thyroid cancers^17, 33^.

Oncocytic (Hürthle cell) tumors have a low avidity for RAI, which poses difficulties for imaging and remnant ablation^30^. Lutje *et al*. found that Hürthle cell carcinoma in their set had decreased uptake of PSMA radiotracer compared to non-oncocytic tumors^33^. Our immunohistochemical study confirmed that oncocytic FA and FTC have significantly lower PSMA expression in neovasculature (in terms of score and frequency) than their non-oncocytic counterparts. This should be kept in mind when interpreting oncocytic thyroid tumors with PSMA imaging.

All kinds of thyroid tumors displayed high variability of endothelial PSMA expression (median score of positive tumors was 30%), which could be easily appreciated on serial sections stained with the broad endothelial markers CD31 and CD34 (Fig. 3A,C). Our initial assumption that PSMA-positive neovasculature can be represented by proliferative endothelium was not confirmed after evaluation with CD105 immunostaining. In fact, CD105 proportion score was significantly higher than PSMA score (P < 0.01, t-test), which means that PSMA expression is not directly related to endothelial cell proliferation. The exact reason for PSMA intratumoral heterogeneity is not clear and needs further investigation.

Evaluation of PSMA immunostaining in our cohort of non-thyroid cancers confirmed that endothelial expression is not organ-specific, but rather universally cancer-specific. Endothelial PSMA expression in various solid cancers was highly heterogeneous in the same fashion as that in thyroid tumors. Detection of PSMA radiotracer-positive foci in the thyroid does not guarantee thyroid origin of these lesions. Recently, a case report has been presented of ^68^Ga-PSMA-avid intrathyroidal metastasis of renal cell carcinoma^22^.

It should be noted that in addition to relatively low sensitivity, the specificity of PSMA expression is not limited exclusively to tumors. Immunohistochemical studies reported PSMA expression in non-neoplastic regenerative tissue, ganglionic cells, and microvessels of tuberculosis lesions^34–36^. Here, we describe for the first time frequent PSMA expression in dendritic cells of lymphoid follicles in Hashimoto’s thyroiditis and cervical lymph nodes. Very recently, Kirchner *et al*. reported incidental uptake of ^68^Ga-PSMA by the thyroid gland in 22% of patients with urological cancers^37^. Considering described diffuse pattern of radiotracer uptake and our findings, inflammatory origin could be a possible reason. Another biodistribution study of ^68^Ga-PSMA found occasional nodal uptake in axillary lymph nodes^32^. PSMA tracer positivity in lymph nodes may present serious pitfall as a mimic of metastasis.

There is a limitation of this study that needs further explanation. We had no opportunity to perform PSMA imaging in our settings; hence we were not able to validate our data by direct correlation between imaging and immunostaining. Nevertheless, there are several independent arguments that could ascertain our findings. Recently, prostate cancer studies found correlation between PSMA tracer uptake and immunohistochemical expression of PSMA^38, 39^. It is important that for correlation purposes PSMA antibody clone 3E6 (the same as in our study) should be used^36^. This clone (unlike another available clone 7E11) binds epitope in the extracellular portion of the PSMA, and all the current PSMA ligands for imaging are based on monoclonal antibodies targeting the extracellular domain of PSMA^20, 40, 41^. Another clinical report supporting our results, found that PSMA tracer uptake in FA was attributed to PSMA immunoreactivity in tumor microvessels, but not in neoplastic epithelium^24^. To sum up, we believe that our main conclusions are valid and may present important background for prospective clinical correlations. It is essential to form a bridge between immunohistochemical and imaging findings in future studies.

Our large scale immunohistochemical study provides rationale for PSMA-targeted imaging and theranostic approaches in thyroid cancer. PSMA is frequently expressed in microvasculature of thyroid tumors, but not in benign thyroid. Endothelial expression is most likely responsible for radiotracer uptake on PSMA imaging of neoplastic thyroid nodules. Significant translational implications are that RAI-refractory tumors and high-grade cancers maintain high level of PSMA expression, and may be targeted by PSMA ligand radiopharmaceuticals. At the same time, our study predicts several pitfalls potentially associated with PSMA imaging of the thyroid, such as absence of organ specificity (wide range of non-thyroid malignancies express PSMA in microvessels), and PSMA-positivity in dendritic cells of chronic thyroiditis and lymph nodes described for the first time. The results presented in this study indicate that ^68^Ga-PSMA radioligand therapy may not be considered specific for thyroid cancer, however targeting of tumor-associated endothelium via PSMA can be a promising strategy for imaging and treatment of RAI-refractory thyroid carcinoma.

## Methods

### Patient cohorts

All samples were collected from archives of the Department of Pathology, King Chulalongkorn Memorial Hospital. The histopathological diagnosis of each case was verified independently by two pathologists with thyroid expertise (AB, SK). Clinicopathological information was retrieved from pathology records. All tumors were staged according to AJCC/UICC TNM staging system^30^. Well-differentiated cancers, such as primary PTC and FTC, were additionally scored according to the MACIS system^30^.

Main cohort was largely reproduced from our recent biomarker study^42^, added by 17 oncocytic thyroid carcinomas and 24 lymph node metastases of papillary thyroid cancer. The final main cohort included 243 thyroid tumors and 191 non-neoplastic samples. Thyroid tumors were FA (n = 43), FTC (n = 52), PTC (n = 120), PDTC (n = 8), ATC (n = 10), and MTC (n = 10). All PTC were subdivided into PTC classic variant (n = 49), PTC follicular variant (n = 33), PTC tall cell variant (n = 5), and papillary microcarcinoma (n = 9), according to the WHO classification^43^. Non-neoplastic samples included histologically unremarkable thyroid (n = 127), nodular goiter (n = 20), Hashimoto’s thyroiditis (n = 30), and Graves’ disease (n = 14). Detailed clinicopathological information is available from our previous report^42^.

Independent cohort comprised 24 cases of RAI-refractory PTC. A case was considered RAI-refractory if the patient had an elevated serum thyroglobulin (>10 ng/ml) under a high thyrotropin level with structural disease in the setting of a negative RAI diagnostic whole-body scan, or metastatic disease progressed after receiving more than 600 mCi of RAI^44^. Inclusion criteria were as follows: RAI total body scan should be performed within 18 months before surgery, and clinically manifested recurrence should be validated by elevated serum thyroglobulin within 18 months before surgery. Structural disease (locoregional metastatic or recurrent) was confirmed on surgical excision with histopathological examination. Exclusion criteria included high-grade thyroid cancers (PDTC, ATC), missing clinical data or loss to follow up, and samples of inadequate size not suitable for immunohistochemical study. Out of 412 cases of well-differentiated thyroid cancer treated and followed up at the Division of Nuclear Medicine, Department of Radiology, Faculty of Medicine, Chulalongkorn University during 2004–2010, 15 recurrent and 9 metastatic PTC were qualified as eligible as RAI-refractory cohort. These were tumors from 21 female and 3 male patients with a mean age of 59.7 ± 14.4 years (range 39–88). There was no overlap between RAI-refractory and main cohorts.

The last cohort in this study included 25 cases of common non-thyroid solid cancers. These were lung adenocarcinoma (n = 3), invasive ductal carcinoma of breast (n = 4), ovarian serous cystadenocarcinoma (n = 1), adenocarcinoma of endometrium (n = 1), stomach (n = 2), colon (n = 4), and pancreas (n = 2), hepatocellular carcinoma (n = 1), renal cell carcinoma (n = 3), and adenocarcinoma of prostate (n = 4).

This study was approved by the Institutional Review Board of the Faculty of Medicine, Chulalongkorn University (Certificate of Approval No. 439/2016). Informed consent was obtained from all subjects. All experiments were carried out in accordance with the approved study plan and relevant guidelines.

### Immunohistochemistry

All immunohistochemical staining was performed on formalin fixed, paraffin embedded whole tissue sections using a Dako Autostainer Link 48 (Dako North America Inc., USA) or Ventana BenchMark system (Ventana Medical Systems, USA) automated immunostainers. Tissue blocks containing the most representative and well-preserved tumor areas were selected for immunostaining. Sections 2 mm thick were positioned on positively charged slides (SuperFrost Plus; Menzer-Glaser, Germany), dewaxed in xylene, and rehydrated using graded alcohols. Table 4 lists the manufacturer, type of antigen retrieval, dilutions, and incubation times for the antibodies used in this study: PSMA as the main marker, and ancillary endothelial markers CD31, CD34, and CD105. PSMA immunostaining was done on all the samples. Immunohistochemistry with endothelial markers was performed for correlation purpose on 50 cases from the main cohort, which showed a wide range of PSMA expression (from negative to strongly positive).

**Table 4:** Antibodies used in the study.

Antibody	Vendor	Clone or ID	Platform	Pre-treatment	Dilution
PSMA	Dako	clone 3E6	Dako	EnVision FLEX™ High pH, 20′ at 95 °C	Pre-diluted, 20′ at RT
CD31	Dako	clone JC70A	Dako	EnVision FLEX™ High pH, 20′ at 95 °C	Pre-diluted, 20′ at RT
CD34	Dako	clone QBEnd 10	Dako	EnVision FLEX™ High pH, 20′ at 95 °C	Pre-diluted, 30′ at RT
CD105	Abcam	ab170943	Ventana	CC1 mild, 30′ at 95 °C	1:500, 32′ at 37 °C

There are two clones of anti-PSMA antibody most commonly used for immunohistochemistry. Clone 7E11 targets an intracellular epitope. Whereas clone 3E6 recognizes an epitope present in the extracellular portion of the PSMA^41^. A point worth making is that only monoclonal antibodies targeting the extracellular domain of PSMA can bind living cells and be effectively used as carriers for imaging and therapeutic agents^40^. In line with this observation, we aimed to check an expression of PSMA by applying anti-PSMA clone 3E6 monoclonal antibody. CD31 and CD34 are pan-endothelial markers, which stain almost all blood vessels. There is minor difference between the two markers in terms of recognizing sinuses of lymph nodes and some stromal cells^45^. CD105, also known as endoglin, is a proliferation-associated endothelial marker expressed in the active dividing endothelial cells of microvessels in cancer tissue^46^.

Benign prostate and prostate adenocarcinoma samples were employed as positive controls for PSMA (cytoplasmic staining of epithelial cells). Capillaries of benign thyroid and skin hemangioma were considered as positive controls for CD31 and CD34 (membranous expression of endothelium). Microvessels of prostate adenocarcinoma served as positive control for CD105 (membranous expression of endothelium)^46^. All positive control samples and random thyroid cancer sections (n = 5) with omitted primary antibody were used as a negative control for all the immunostains used. All microphotographs were acquired using Olympus BX53 F microscope equipped with Olympus DP26 high-resolution microscope camera (Olympus System, Japan).

### Evaluation of immunostaining with PSMA and endothelial markers

Positive PSMA expression was defined as cytoplasmic immunoreactivity in epithelial (benign or neoplastic) cells, or membranous immunoreactivity in endothelial cells lining vessels. Initially immunoexpression of PSMA in vascular endothelium was semi-quantitatively scored based on intensity (negative, weak, moderate, and strong) and proportion score (in 5% increments). After recognizing that staining intensity directly correlates with the extent of staining in tumor-associated vessels, similar to the previous study of Wernicke *et al*. on breast cancer, the proportion score was chosen as the most appropriate and easier to replicate output of PSMA scoring^9^. We considered 5% threshold for a binary model of PSMA scoring (positive or negative) based on the study, which found that around 5% of PSMA-stained microvessels in thyroid adenoma were able to produce marked accumulation of ^68^Ga-PSMA ligand on PET/CT imaging^24^. Furthermore, for easier reproduction of our results in potential future studies, PSMA score in vascular endothelium was simplified to a three-tiered scale: score 0 for tissues with no detectable endothelial PSMA expression and incidental expression in <5% of capillaries; score 1 for tumors with PSMA expression in 5–50% of microvessels; and score 2 for tumors that showed PSMA expression in >50% of microvessels^9^.

A training set of 50 samples including different thyroid lesions from the main cohort was employed to adjust a reader’s skills to correctly identify microvascular network, and to perform precise PSMA scoring. For this reason, all 50 samples were immunostained with broad endothelial markers CD31 and CD34. Matched PSMA, CD31, and CD34 slides were digitized by an Aperio CS2 slide scanner (Leica Biosystems, USA), and automatically scored using the Aperio Imagescope v.10.2.2.2352 software with commercially available Microvessel Analysis v.1 algorithm (Aperio Technologies, USA). Practicing with the training set could achieve excellent concordance between manual and automated scores, hence manual scoring was selected as a feasible and time saving approach for the study.

A procedure of PSMA scoring in microvessels was set as follows: review of corresponding H&E and PSMA immunostained slides, separate evaluation of the microvascular network in benign and neoplastic tissues, detection of PSMA-positive capillaries (on low and high magnification), and record proportion and intensity scores into the spreadsheet. Medium- and large-caliber vessels were excluded. Two pathologists (AB, SK) performed independent evaluations, and the final score was recorded as a mean of two. Cases significantly discordant between the two readers were resolved by consensus review.

### Statistical analysis

All data were entered into Microsoft Office Excel 2007 spreadsheet software (Microsoft Corporation, USA). All statistical analyses were performed using SPSS Statistics version 17.0 (SPSS IBM, USA). Numerical data were evaluated for normality by Shapiro-Wilks and Kolmogorov-Smirnov tests. Student’s t-test, Mann-Whitney U test, and Kruskal-Wallis test were used to compare continuous variables. Pearson correlation coefficient or Spearman’s correlation coefficient (nonparametric) were used to determine the relationship between immunostaining scores and clinical variables. Fisher’s exact test or chi square test were used to compare categorical variables. A two-sided p value of less than 0.05 was considered statistically significant.

### Data availability

The datasets generated during and/or analyzed during the current study are available from the corresponding author on reasonable request.
